# Long noncoding RNA MALAT1 promotes malignant development of esophageal squamous cell carcinoma by targeting β-catenin *via* Ezh2

**DOI:** 10.18632/oncotarget.8257

**Published:** 2016-03-22

**Authors:** Wei Wang, Yunan Zhu, Sanni Li, Xinfeng Chen, Guozhong Jiang, Zhibo Shen, Yamin Qiao, Liping Wang, Pengyuan Zheng, Yi Zhang

**Affiliations:** ^1^ Department of Oncology, The First Affiliated Hospital of Zhengzhou University, Zhengzhou, Henan 450052, China; ^2^ The Third People's Hospital of Zhengzhou, Zhengzhou, Henan 450000, China; ^3^ Biotherapy Center, The First Affiliated Hospital of Zhengzhou University, Zhengzhou, Henan 450052, China; ^4^ Department of Pathology, The First Affiliated Hospital of Zhengzhou University, Zhengzhou, Henan 450052, China; ^5^ Engineering Key Laboratory for Cell Therapy of Henan Province, Zhengzhou, Henan 450052, China; ^6^ Department of Gastroenterology, The Fifth Affiliated Hospital of Zhengzhou University, Zhengzhou, Henan 470000, China; ^7^ School of Life Sciences, Zhengzhou University, Zhengzhou, Henan 450001, China

**Keywords:** ESCC, lncrna, MALAT1, β-catenin, Ezh2

## Abstract

Evidences have shown that lncRNAs involve in the initiation and progression of various cancers including esophageal squamous cell carcinoma (ESCC). The aberrant expression of lncRNA MALAT1 was investigated in 106 paired ESCC tissues and adjacent non-cancerous tissues by qRT-PCR. Down-regulated MALAT1 and Ezh2 over-expression plasmid were constructed respectively to analyze the expression of β-catenin, Lin28 and Ezh2 genes. We found that the MALAT1 expression level was higher in human ESCC tissues (*P*=0.0011), which was closely correlated with WHO grade (*P*=0.0395, *P*=0.0331), lymph node metastasis (*P*=0.0213) and prognosis (*P*=0.0294). Silencing of MALAT1 expression inhibited cell proliferation, migration and tumor sphere formation, while increasing cell apoptosis of esophageal cancer *in vitro*. Down-regulation of MALAT1 decreased the expression of β-catenin, Lin28 and Ezh2 genes, while over-expressed Ezh2 combined with MALAT1 down-regulation completely reversed the si-MALAT1-mediated repression of β-catenin and Lin28 in esophageal cancer cells. Animal experiments showed that knockdown of MALAT1 decreased tumor formation and improved survival. MALAT1 promotes the initiation and progression of ESCC, suggesting that inhibition of MALAT1 might be a potential target for treatment of ESCC.

## INTRODUCTION

Esophageal cancer (EC) is one kind of the most frequently invasive malignancies and ranks as the sixth cancer-related mortality in worldwide [1]. Here, an urgent problem is how to provide more effective therapeutic strategies to treat the patients with ESCC. More and more evidences have shown that the long noncoding RNAs (lncRNAs) are multiple biomarkers for predicting cancer in recent years [2]. Exploring the aberrant expression of the lncRNAs contributes to a better understanding of the molecular events and offers insight into potential molecular targets for diagnosis and therapy in ESCC [3].

Metastasis-associated lung adenocarcinoma transcript 1 (*MALAT*1) is a bona fide lncRNA [4]. Increasing evidences have shown that the aberrant expression of MALAT1 plays a crucial role in cancinogenesis [5]. MALAT1 was firstly reported in invasive non-small cell lung carcinoma, over-expression of MALAT1 was investigated in the cancers of liver [6], colon [7], cervical [8], etc. However, the function of MALAT1 and molecular mechanism in ESCC remains largely unknown. β-catenin is a most important linchpin of Wnt/β-catenin pathway for regulation of cell proliferation, differentiation, migration, and malignant occurrence [9, 10]. Accumulated evidences have shown that β-catenin is activated in many cancers including ESCC [11, 12]. However, whether MALAT1 participates in the aberrant activation of β-catenin and related molecular mechanism involved in ESCC is still poor [13].

The purpose of the study was to investigate the expression and function of MALAT1 in ESCC. We also tried to understand the relationship between MALAT1, β-catenin and Ezh2 in ESCC, and seek the cellular and molecular mechanisms of the occurrence and progression of ESCC and find a potential therapeutic target for ESCC.

## RESULTS

### Cell lines screening

To understand the effect of MALAT1 in the progression of ESCC, we detected the expression level of MALAT1 by quantitative real time PCR (qRT-PCR) in six human ESCC cell lines TE1, TE7, EC1, EC109, KYSE70 and KYSE450. We found that TE7 cells expressed the highest mRNA level of MALAT1 (*P*<0.0001, Supplementary Figure S1).

### Relationship between MALAT1 expression and clinic pathological characteristics in ESCC patients

**Figure 1:**
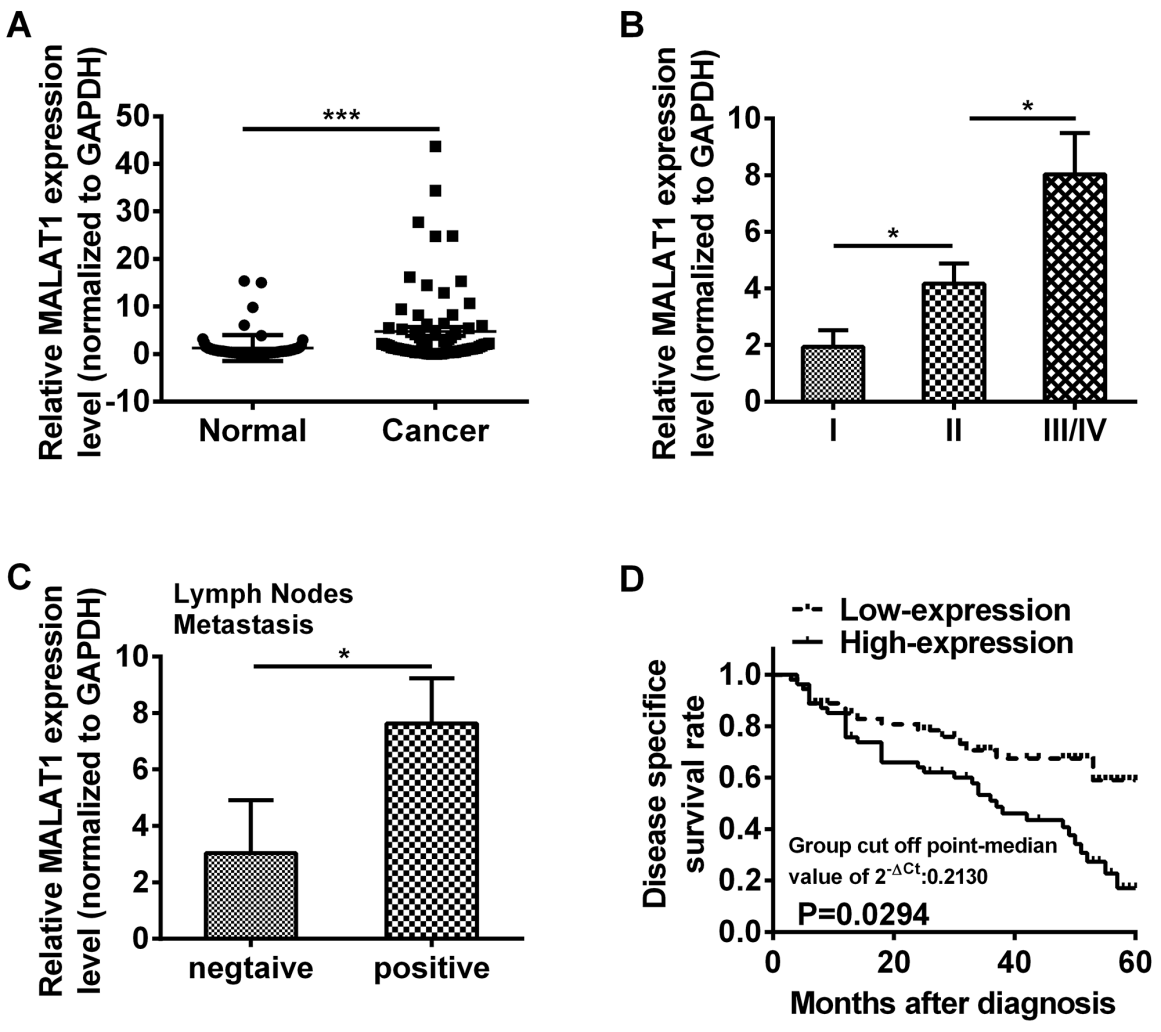
LncRNA MALAT1 was over-expressed in ESCC tissues **A.** Relative expression of MALAT1 was measured by qRT-PCR in 106 ESCC tissues and matched adjacent non-cancerous tissues. The relative value of gene expression was normalized against the gene expression levels of GAPDH. **B.** The expression of MALAT1 was significantly higher in patients at advanced pathological stages. **C.** The expression of MALAT1 was compared in ESCC patients with or without lymph nodes metastasis. **D.** Kaplan-Meier overall survival curves by MALAT1 expression level. Patients with MALAT1 high-expression (n=46) had a significantly poorer overall survival than those with low-expression (n=60). Data represent the mean ± SD. *, *P*<0.05, ***, *P*< 0.001.

To investigate the role of MALAT1 in ESCC, 106 fresh tissue samples of ESCC and matched adjacent non-cancerous tissues were detected by qRT-PCR, respectively. The MALAT1 expression level was notably higher in cancer tissues compared with that in matched adjacent non-cancerous tissues (*P*=0.0011, Figure 1A, Table 1). In order to explore the association between MALAT1 expression and clinic pathological characteristics, 106 patient tissues with ESCC were analyzed. We found that the MALAT1 expression level was gradually increased in ESCC patients in line with WHO stage (*P*=0.0395, *P*=0.0331, Figure 1B, Table 2) and revealed that MALAT1 was predominantly up-regulated in late-stage tumor tissues. Additionally, the MALAT1 expression level was closely associated with lymph nodes metastasis (*P*=0.0213, Figure 1C, Table 2), while no association was found between the expression of MALAT1 and gender, age in ESCC (Table 2).

**Table 1:** The expression of MALAT1 in ESCC tissues and adjacent non-cancerous tissues

Tissues	Relative MALAT1 Expression level	*P* value
non-cancerous	1.586±0.5943	
ESCC	5.883±2.166	0.0011

Continuous variable data are given as mean±SD (range). The relative value of gene expression was normalized against the gene expression levels of GAPDH.

**Table 2:** Correlation between MALAT1 expression and clinical parameters in ESCC tissues

Clinical parameters	Relative MALAT1 expression level in cancer tissues	*P* value
***Gender***		
Male	6.859±4.856	
Female	7.601±4.469	0.9118
***Age***		
>60	6.513±4.338	
<60	7.804±4.804	0.8482
***Lymph nodes***		
***metastasis***		
N	3.041±0.5924	
Y	7.630±1.597	0.0213
***Clicinal stage***		
I	1.937±0.5879	
II	4.173±0.7093	0.0395
III/IV	8.025±1.468	0.0331

Continuous variable data are given as mean±SD (range). The relative value of gene expression was normalized against the gene expression levels of GAPDH.

To further explore the prognostic value of MALAT1, we divided the patients with ESCC into two groups according to MALAT1 expression levels (high-expression and low-expression) and assessed the association between the MALAT1 expression level and overall survival through Kaplan-Meier analysis and log-rank test. Results revealed that patients with MALAT1 high-expression level had a significantly poorer overall survival than those with low-expression level (*P*=0.0294, Figure 1D).

### MALAT1 down-regulation decreased cancer stem cell-like traits *in vitro*

**Figure 2:**
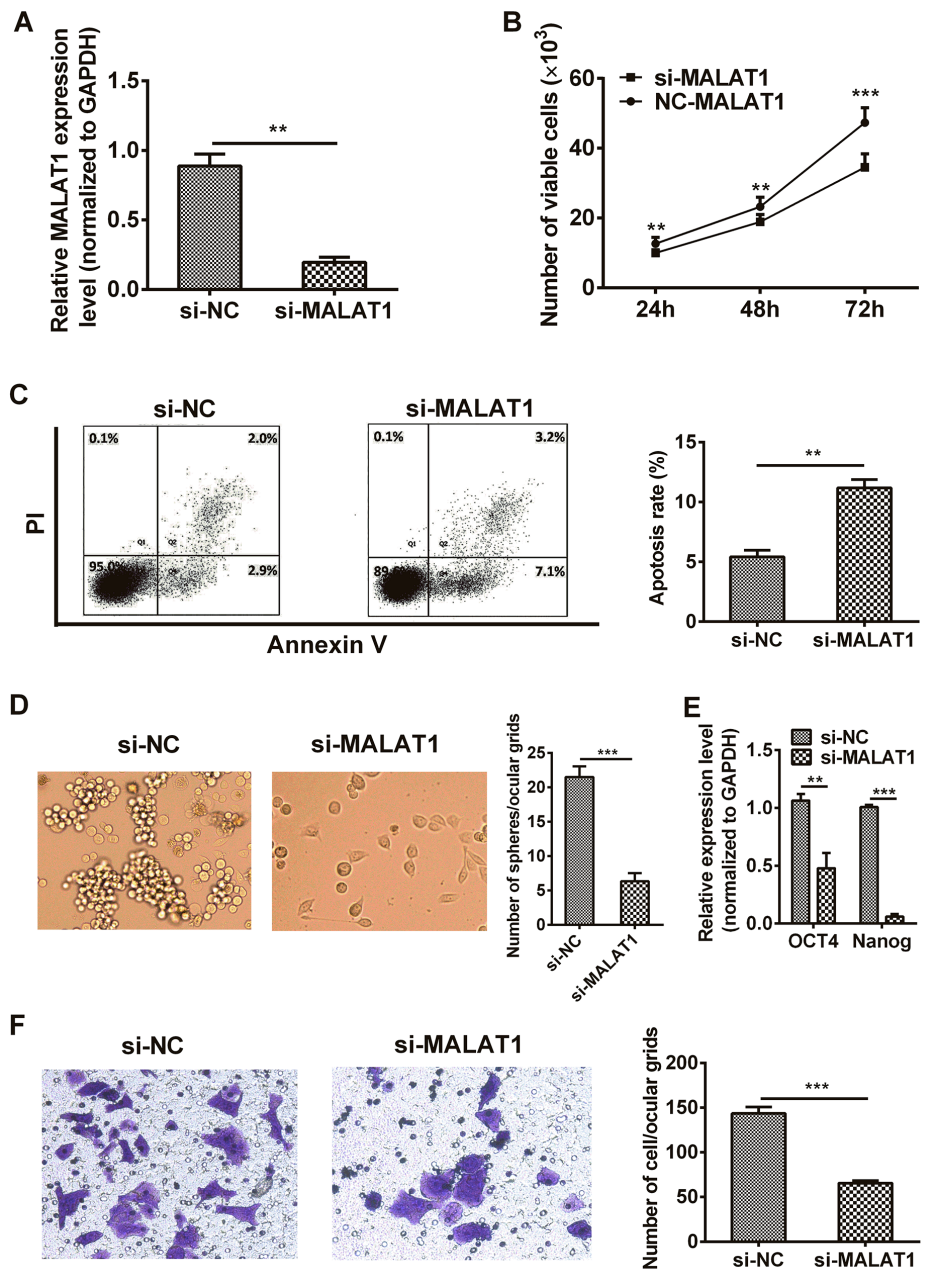
Down-regulation of MALAT1 inhibited malignant potential in TE7 cells **A.** The MALAT1 mRNA expression level in TE7 cells transfected with si-MALAT1 or si-NC were measured by qRT-PCR. **B.** MALAT1 down-regulation suppressed the proliferation of TE7 cells *in vitro*. CCK-8 assay was performed to determine cells growth. **C.** The percentage of apoptotic cells transfected with si-MALAT1 or si-NC were detected by flow cytometric analysis of annexin V/PI staining. Apoptosis rate was assessed by counting the percentage of early apoptotic and late apoptotic cells. **D.** Cells transfected with si-MALAT1 formed fewer, smaller mammospheres. Representative photographs of mammospheres were taken at 7 day (left) (original magnification, 50×), the number of spheres was quantified (right). **E.** EMT stem gene (eg: OCT4 and Nanog) expressions were detected by qRT-PCR after MALAT1-siRNA. **F.** TE7 cells transfected with si-MALAT1 displayed significantly lower transmembrane migration capacity compared with those transfected with si-NC (left) (original magnification, 100×). The bar chart represented the number of cells migrated into the lower chambers (right). Data are representative of three independent experiments and represent the mean ± SD. **, *P*< 0.01, ***, *P*< 0.001.

MALAT1 specific siRNA (si-MALAT1) and non-specific siRNA used as negative control (si-NC) were transfected into TE7 cells, respectively. As shown in Figure 2A, cells transfected with si-MALAT1 showed a significant decreased (more than 70%) mRNA expression of MALAT1 compared to the si-NC group (*P*=0.0015). CCK-8 assays revealed that cell growth was suppressed in TE7 cells transfected with si-MALAT1 compared with si-NC group (24 hr: *P* = 0.0011, 48 hr: *P*=0.0015, 72 hr *P*=0.0002, Figure 2B). Apoptotic rate of cells transfected with si-MALAT1 was notably elevated compared with si-NC group (*P*=0.0043, Figure 2C). Sphere formation assay also revealed that cells transfected with si-MALAT1 formed fewer and smaller spheres than si-NC group (*P*=0.0008, Figure 2D). We also investigated the effect of tumor stemness genes after cells transfected with si-MALAT1 by qRT-PCR, we found that silencing MALAT1 down-regulated the expression of OCT4 and Nanog genes (*P*=0.0022, *P*=0.0005, Figure 2E). In addition, transwell assay was performed to analyze the role of MALAT1 in cell migration, results presented that TE7 cells transfected with si-MALAT1 was distinctively less migratory than the cells transfected with si-NC (*P*=0.0005, Figure 2F).

### The effect of MALAT1 on β-catenin, E-cadherin, Lin28, OCT4 and Ezh2 in esophageal cancer cells

**Figure 3:**
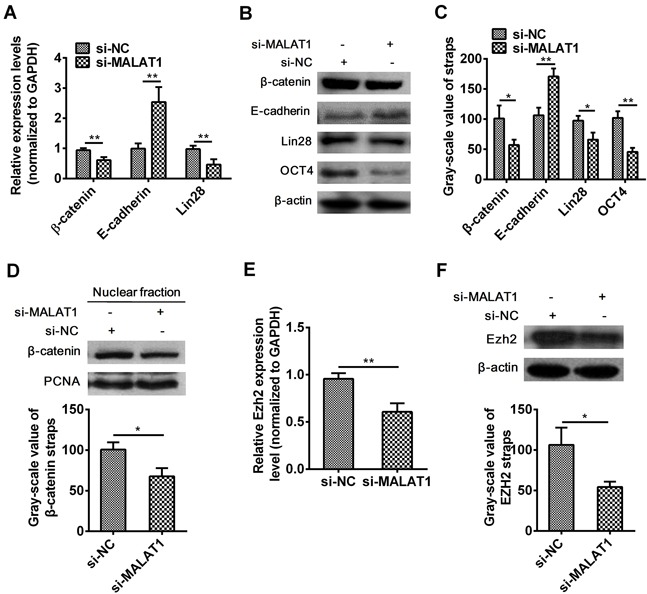
Effect of MALAT1 down-regulation on β-catenin, E-cadherin, Lin28, OCT4 and Ezh2 expressions **A.** The expression of β-catenin, E-cadherin, Lin28 were detected in cells transfected with si-MALAT1 or si-NC by qRT-PCR. **B.** MALAT1 down-regulation suppressed the protein expressions of β-catenin, E-cadherin, Lin28, OCT4 by Western blots. **C.** Western blots Data were representative of three independent experiments and represent the mean ± SD. **D.** Nuclear localization of β-catenin was assessed in ESCC after si-MALAT1 by Western blots. **E.** Ezh2 mRNA expression level was detected by qRT-PCR after MALAT1 down-regulation. **F.** Ezh2 protein expression level was detected by Western blots after MALAT1 down-regulation. Data are representative of three independent experiments and represent the mean ± SD. **, *P*< 0.01.

It was reported that resveratrol decreased nuclear localization of β-catenin thus attenuated β-catenin signaling after silencing MALAT1 in colorectal cancer cells [36]. To investigate the explicit molecular mechanism of MALAT1 on the initiation and progression of ESCC,β-catenin, E-cadherin, Lin28, OCT4 and Ezh2 expressions were examined. As expected, the mRNA level of β-catenin (*P*=0.0014, Figure 3A) and Lin28 (*P*=0.0026, Figure 3A) and the protein level of β-catenin (*P*=0.0311, Figure 3B), Lin28 (*P*=0.0178, Figure 3B) and OCT4 (*P*=0.0016, Figure 3B) were significantly decreased and E-cadherin mRNA (*P*=0.0011, Figure 3A) and protein (*P*=0.0037, Figure 3B) expression level were dramatically increased in si-MALAT1-treated esophageal cancer cells. To investigated if β-catenin located in tumor cell nucleus, we detected the nuclear fraction of β-catenin by Western blots and found that nucleus β-catenin was also notably decreased (*P*=0.0139, Figure 3D). The above results indicated that MALAT1 regulated the expression of β-catenin, E-cadherin, Lin28 and OCT4 genes in ESCC.

Evidences have shown that, E-cadherin expression was increased, whereas β-catenin expression was decreased through enhancer of zeste homolog 2 (Ezh2) after silencing MALAT1 [38]. Results illustrated that down-regulation of MALAT1 inhibited the expression level of Ezh2 from mRNA (*P*=0.0012, Figure 3E) to protein (*P*=0.0155, Figure 3F) in TE7 cells.

### Association of Ezh2 expression and clinic pathologic parameters in ESCC tissues

**Figure 4:**
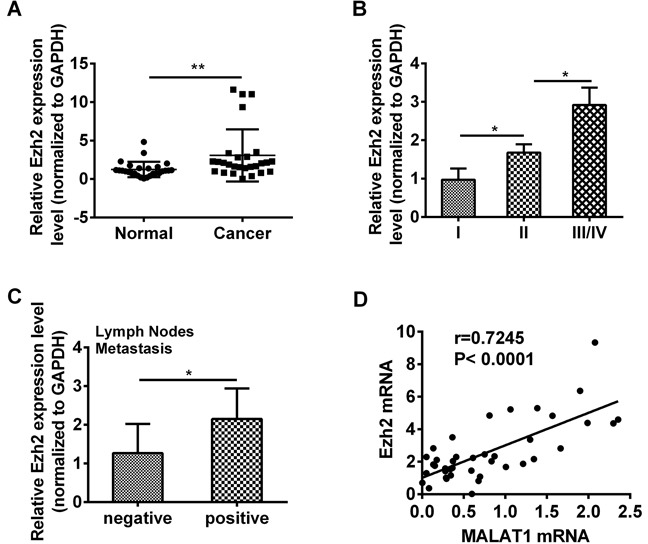
Ezh2 expression was up-regulated in ESCC tissues **A.** Relative expression of Ezh2 was measured by qRT-PCR in 42 ESCC tissues and matched adjacent non-cancerous tissues. The relative value of gene expression was normalized against the gene expression levels of GAPDH. **B.** The expression of Ezh2 was significantly higher in patients with advanced pathological stages. **C.** The expression of Ezh2 was analyzed in ESCC patients with or without lymph nodes metastasis. **D.** There was a significant positive correlation between MALAT1 and Ezh2 in ESCC tissues (R=0.7245, *P*<0.0001). Each symbol indicated one patient and the line represented the regression line. Data represent the mean ±SD. *, *P*<0.05, **, *P*< 0.01.

Given that MALAT1 modulated Ezh2 expression in esophageal cancer cells, then we examined the expression of Ezh2 in 40 ESCC tissues. We found that the Ezh2 expression level was notably elevated in cancer tissues compared with that in matched adjacent non-cancerous tissues (*P*=0.0024, Figure 4A, Table 3). As shown in Table 4, the Ezh2 expression level was elevated in ESCC patients in line with WHO stage and predominantly up-regulated in late-stage tumor tissues (*P*=0.0221, *P*=0.0302, Figure 4B, Table 4). Additionally, Ezh2 expression level was closely associated with lymph node metastasis (*P*=0.0124, Figure 4C). We compared the mRNA expression level of MALAT1 and Ezh2 in ESCC cancer tissues and found that there was remarkably positive correlation between MALAT1 and Ezh2 expression (*P*<0.0001, Figure 4D).

**Table 3:** The expression of Ezh2 in ESCC tissues and adjacent non-cancerous tissues

Tissues	Relative Ezh2 Expression level	*P* value
non-cancerous	1.238±0.1923	
ESCC	3.080±0.6515	0.0090

Continuous variable data are given as mean±SD (range). The relative value of gene expression was normalized against the gene expression levels of GAPDH.

**Table 4:** Correlation between Ezh2 expression and clinical parameters in ESCC tissues

Clinical parameters	Relative Ezh2 expression level in cancer tissues	*P* value
***Gender***		
Male	3.602±1.698	
Female	4.072±1.378	0.3250
***Age***		
>60	3.932±1.599	
<60	3.743±1.498	0.9326
***Lymph nodes metastasis***		
Negative	1.269±0.2186	
Positive	2.151±0.2383	0.00124
***Clicinal stage***		
I	0.9719±0.2888	
II	1.854±0.2132	0.0221
III/IV	2.917±0.4561	0.0302

Continuous variable data are given as mean±SD (range). The relative value of gene expression was normalized against the gene expression levels of GAPDH.

### The effect of Ezh2 on the expression of β-catenin and Lin28 in esophageal cancer cells

**Figure 5:**
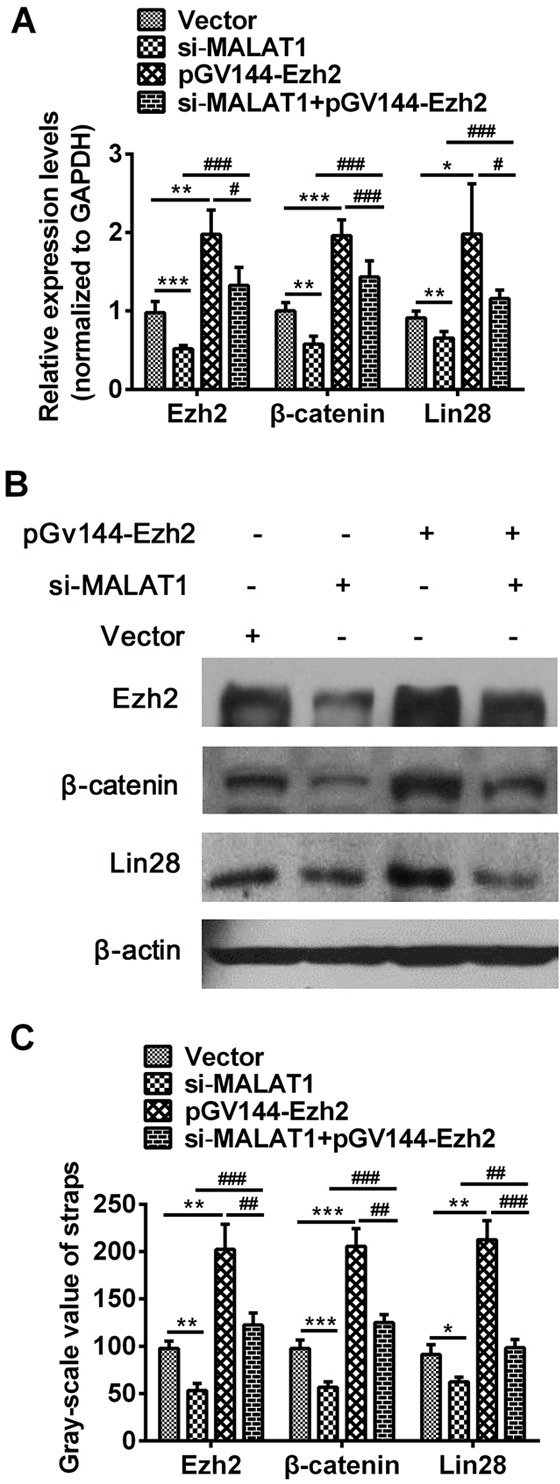
Over-expressed Ezh2 combined with MALAT1 down-regulation completely reversed the si-MALAT1-mediated repression of β-catenin and Lin28 in esophageal cancer cells **A.** The expressions of β-catenin, Lin28 and Ezh2 were measured in cells transfected with si-MALAT1, pGV144-Ezh2, si-MALAT1 +pGV144-Ezh2 or pGV144-NC by qRT-PCR. **B.** β-catenin, Lin28 and Ezh2 expressions in cells with si-MALAT1, pGV144-Ezh2, si-MALAT1+pGV144-Ezh2 or pGV144-NC were detected by Western blots. **C.** Western blots Data were representative of three independent experiments and represent the mean ± SD. *, *P*<0.05, **, *P*< 0.01, ***, *P*< 0.001.

In order to verify the effect of Ezh2 on β-catenin and Lin28 genes, over-expression Ezh2 recombinant plasmid pGV144-Ezh2 was constructed. The results indicated that over-expression of Ezh2 was significant increased (more than 80%) compared to cells that were transfected with NC (*P*=0.0018, Supplementary Figure S2A and *P*=0.0045, Figure S2C). We had confirmed that down-regulation of MALAT1 inhibited the expression of Ezh2, β-catenin and Lin28, However, as shown in Figure 5, both qRT-PCR (Ezh2: *P*=0.0008, *P*=0.0011, *P*=0.0004, *P*=0.0149, β-catenin: *P*=0.0013, *P*=0.0001, *P*=0.0002, *P*=0.0003, Lin28: *P*=0.0047, *P*=0.0162, *P*=0.0006, *P*=0.0441, Figure 5A) and Western blots (Ezh2: *P*=0.0024, *P*=0.0039, *P*=0.0005, *P*=0.0052, β-catenin: *P*=0.0004, *P*=0.0003, *P*=0.0001, *P*=0.0063, Lin28: *P*=0.0234, *P*=0.0043, *P*=0.0028, *P*=0.0004, Figure 5B) results revealed that over-expressed Ezh2 combined with MALAT1 down-regulation completely reversed the si-MALAT1-mediated repression of β-catenin, Lin28 and Ezh2, indicating MALAT1 promoting malignant development of ESCC by targeting β-catenin via Ezh2.

### Knockdown of MALAT1 expression decreased tumor formation and improved survival *in vivo*

**Figure 6:**
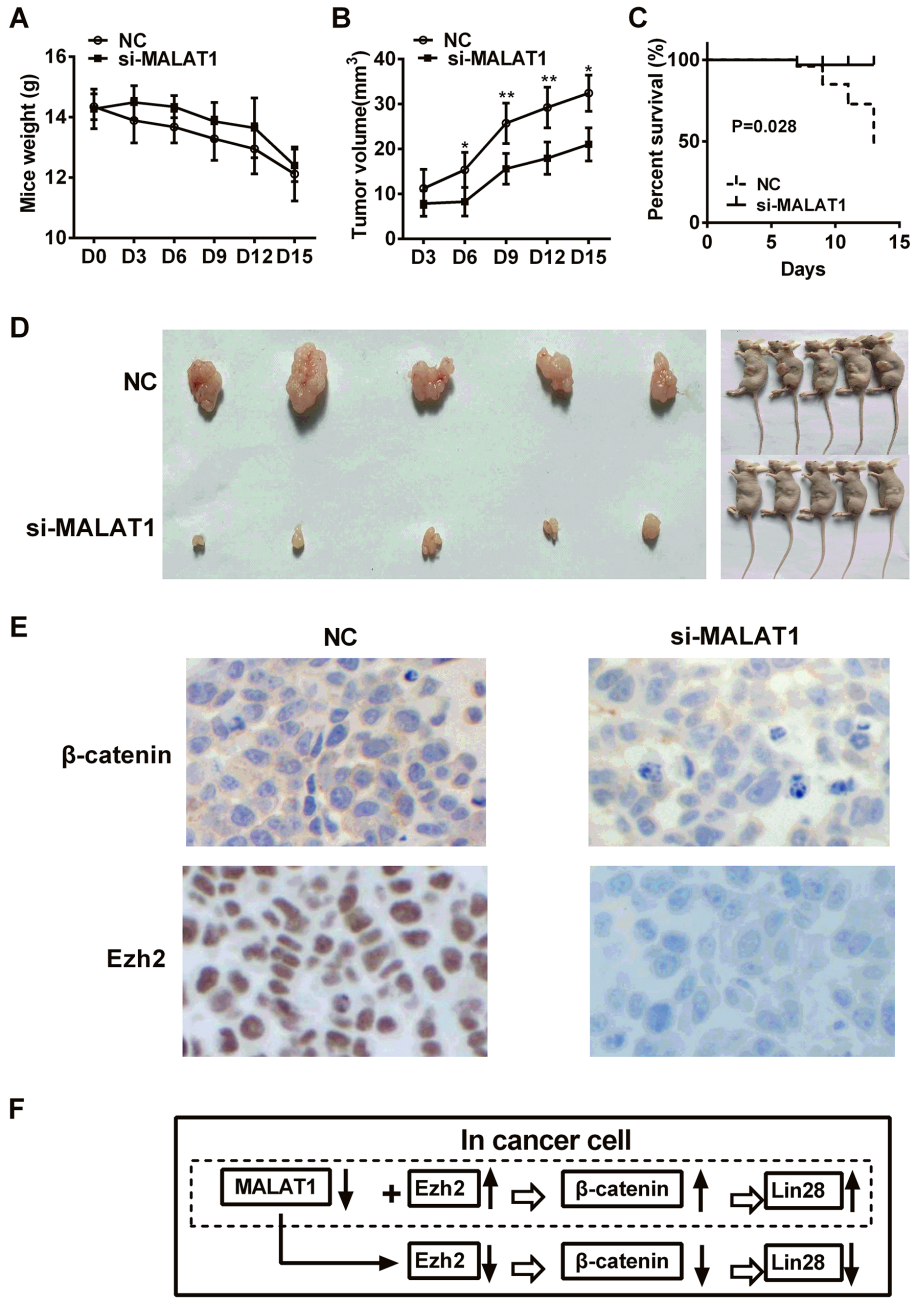
Effect of MALAT1 knockdown on the growth of ESCC *in vivo* **A.** The body weights of mice were detected in MALAT1-shRNA group and control group. **B.** Tumor volume was measured in MALAT1-shRNA group and control group. **C.** Nude mice with negative control group had a significantly poorer overall survival than those with MALAT1-shRNA group (*P*=0.0280). **D.** The images of tumors in nude mice formed by MALAT1-shRNA group and control group stably transfected TE7 cells were shown. **E.** Immunohistochemistry analysis showed that β-catenin expression was distributed in the membrane of cancer cells and significantly decreased in MALAT1-shRNA group compared with control group, and Ezh2 expression demonstrated the lower expression level in cell nucleus after MALAT1 knockdown. **F.** Schematic diagram of MALAT1 regulation β-catenin via Ezh2. We concluded that MALAT1 expression was higher in ESCC tissues than in paired adjacent non-cancerous tissues. MALAT1 down-regulation decreased the expressions of β-catenin, Lin28 and Ezh2. Over-expressed Ezh2 combined with MALAT1 down-regulation completely reversed the si-MALAT1-mediated repression of β-catenin and Lin28 in esophageal cancer cells. Collectively, lncRNA MALAT1 plays a crucial role in the progression of ESCC by regulating the expression of β-catenin and Lin28 via Ezh2. Data were representative of three independent experiments and represent the mean ± SD. *, *P*<0.05, **, *P*< 0.01, ***, *P*< 0.001.

To knock down MALAT1 expression, we used pGV248 vector encoding a small hairpin RNA directed against MALAT1 in TE7 cells. shRNA vector without hairpin oligonucleotides as negative control. There was no significant difference in the body weights of mice between MALAT1-shRNA group and control group (*P*>0.05, Figure 6A), while there were significant differences in tumor volume between MALAT1-shRNA group and control group (*P*<0.05, Figure 6B). Results also revealed that nude mice with negative control group had a significantly poorer overall survival than those with MALAT1-shRNA group (*P*=0.0280, Figure 6C). We also investigated the images of tumors in nude mice formed by MALAT1-shRNA group and control group stably transfected TE7 cells were shown in Figure 6D. Immunohistochemistry analysis showed that β-catenin expression was distributed in the membrane of cancer cells and significantly decreased in MALAT1-shRNA group compared with control group, and Ezh2 expression demonstrated the lower expression level in cell nucleus after MALAT1 knockdown (Figure 6E).

## DISCUSSION

ESCC is the most prevalent histopathologic subtype of EC in central regions of China, especially in Henan province [14]. Despite of the recent rapid advances in the diagnosis and treatment, survival of ESCC patients with recurrence or metastasis remains unfavorable [15, 16]. The improvement of ESCC survival rate requires a clear understanding of pivotal molecular mechanisms from the initiation and progression of ESCC.

LncRNAs represent a new class of non-protein-coding RNA (>200 nt) due to lacking of an intact open reading frame. They have involved in many vital biological activities including epigenetic regulation, transcription and post-transcription, etc [17]. It was reported that lncRNA LOC285194 was associated with larger tumor size, advanced TNM stage, more lymph node metastases and distant metastases [18], etc. Several lncRNAs including Prostate Cancer-Associated ncRNA Transcript 1 (PCAT-1) [19], Urothelial Carcinoma Associated 1 (UCA1) [20] and SPRY4-IT1 [21] were significantly increased in ESCC tissues and their high expressions were significantly correlated with the tumor invasion, advanced clinical stage, lymph node metastasis and poor prognosis. Although progression has been made in understanding the function of lncRNAs, MALAT1 expression pattern and molecular mechanism involved in ESCC remains largely unknown. Dinger et al [22] confirmed that MALAT1 played an important role in the procession of pre-mRNA and recruiting phosphorylated serine/arginie riched protein (SR protein) by MALAT1-associated small cytoplasmic RNA (mascRNA). Garen et al [23] found that the overexpression of MALAT1 reduced tumor suppressor proteins (TSP) and participated in the carcinogenesis. In the present study, we identified that increasing MALAT1 levels in ESCC tissues versus adjacent non-cancerous tissues by qRT-PCR, and the over-expression of MALAT1 associated with clinic pathological parameters, for example, histological grade and lymph node metastasis, as well as survival rate. After MALAT1 specific siRNA (si-MALAT1) was transfected in TE7 cells, we noted that the down-regulation of MALAT1 expression inhibited cell proliferation, migration, tumor sphere formation, while increasing esophageal cancer cell apoptosis *in vitro*, as the results reported by Wang et al [24], in which the abilities of migration and invasion of ESCC cells were inhibited after silencing MALAT1.

Currently, it have been verified that MALAT1 is oncogene by post-transcriptional regulation mechanism [25]. MALAT1 is a nuclear long non-coding RNA, whose expression has associated with a migratory phenotype and tumor stem regulation in several cancer types [26-31]. β-catenin pathway is an ancient evolutionary signaling and involves in the regulation of a wide variety of physiological and pathologic processes, including embryogenesis, differentiation, and carcinogenesis [32]. β-catenin participates in cell-cell adhesion and signal transduction as a downstream linchpin of canonical Wnt/β-catenin pathway [33]. We have published that β-catenin pathway was activated in ESCC progression leading to a poor prognosis of ESCC in 2010 [34]. Although great effort has been made in understanding the molecular regulation of β-catenin pathway, little is known about the correlation between MALAT1 and β-catenin in ESCC. Vassallo et al [35] revealed that the Wnt inhibitory factor 1 (WIF1), which was a secreted inhibitor of WNTs, suppressed the expression of MALAT1 in glioblastoma and loss of WIF1 enhanced the migratory potential of glioblastoma through Wnt5A that activated the Wnt/Ca^2+^ pathway and MALAT1. Ji et al [36] investigated that resveratrol down-regulated MALAT1, resulting in decreased nuclear localization of β-catenin thus attenuated Wnt/β-catenin signaling, which led to the inhibition of invasion and metastasis of colorectal cancer cells. In our report, we found that down-regulation of MALAT1 notably declined the expression of β-catenin, Lin28, Ezh2 and EMT stem genes, such as OCT4 and Nanog, while increased the E-cadherin expression level.

It is well known that methyl-transferase enhancer of zeste homolog 2 (Ezh2), the catalytic activity subunit of Polycomb Repressive Complex 2 (PRC2), usually acts as closing gene function. MALAT1 has been reported to bind to zeste12 inhibitor Suz12, which is a component of PRC2 [37]. Hirata et al [38] elucidate that MALAT1 was transcriptionally activated by c-Fos and interacted with Ezh2. After MALAT1 silencing, E-cadherin expression was increased, whereas β-catenin expression was decreased through Ezh2. Ren et al [39] revealed that Ezh2 accelerated by inhibiting tumor cell invasion via inhibiting RKIP and Ezh2 regulated RKIP transcription, it may be a new mechanism Ezh2 stimulating tumor progression and metastasis. Fillmore et al [40] revealed that Ezh2 inhibition had differential effects on the TopoII inhibitor response of non-small-cell lung cancers both *in vitro* and *in vivo*. Béguelin et al [41] reported that Ezh2 inhibition generated huge synergy role combined with BCL2 inhibition in diffuse large B cell lymphomas (DLBCLs). In this report, we found Ezh2 expression was significantly higher in ESCC tissues compared with normal tissues and there was obvious correlation between MALAT1 and Ezh2 expression in ESCC. Additionally, increased expression level of Ezh2 speeded the progress of ESCC, which not only reflected in the gradual elevation of Ezh2 expression from normal esophagus to cancer, but also in the relationship of the Ezh2 expression and WHO grades and lymph node metastasis. Lastly, over-expressed Ezh2 combined with MALAT1 down-regulation completely reversed the si-MALAT1-mediated repression of β-catenin and Lin28 in esophageal cancer cells. Based on results above, we believed that Ezh2 not only involved in the carcinogenic process, but also activated in tumor progression.

In conclusion, our findings demonstrate that LncRNA MALAT1 plays a crucial role in the progression of ESCC by regulating the expression of β-catenin and Lin28 via Ezh2, indicating inhibition of MALAT1 might be a potential target for treatment of ESCC.

## MATERIALS AND METHODS

### Tissues specimens

106 paired ESCC tissues and adjacent non-cancerous tissues were respectively collected in the First Affiliated Hospital of Zhengzhou University from 2011 to 2014. ESCC tissues and adjacent non-cancerous tissues were obtained from the resected tumors and adjacent non-cancerous esophagus, respectively. None of the patients had undergone chemotherapy or radiotherapy prior to surgery. All pathological results were confirmed by two senior pathologists.

### qRT-PCR

qRT-PCR were performed using SYBR Green method. Total RNA separated from 1×10^6^ cells or tissue samples by Trizol reagent and was performed with hexamer random primers using the First Strand cDNA Synthesis Kit. The mRNA expression level was quantified in duplicate on the Stratagene Mx3005P (Agilent Technologies). All Primers used for qRT-PCR were listed in Table 5 (GAPDH was used for normalization of data). Each sample obtained from three independent experiments was used for analysis of relative gene expression using the 2^−ΔΔCt^ method.

**Table 5:** Paired Primers were used for qRT-PCR

Genes	Primers	Product (bp)
β-catenin	Forward: 5′-CAACTAAACAGGAAGGGATGGAAGG- 3′ Reward: 5′- CAACTAAACAGGAAGGGATGGAAGG - 3′	240
MALAT1	Forward: 5′- TGCGAGTTGTTCTCCGTCTAT - 3′ Reward: 5′- CTTATCTGCGGTTTCCTCAAG - 3′	119
Lin28	Forward: 5′- CGGGCATCTGTAAGTGGTTC - 3′ Reward: 5′- CAGACCCTTGGCTGACTTCT - 3′	191
Ezh2	Forward: 5′- AGGACGGCTCCTCTAACCAT- 3′ Reward: 5′- CTTGGTGTTGCACTGTGCTT- 3′	179
GAPDH	Forward: 5′- GCTGAGAACGGGAAGCTTGT- 3′ Reward: 5′- GCCAGGGGTGCTAAGCAGTT - 3′	154

### Cell culture

Human esophageal cancer cell lines (TE1, TE7, EC1, EC109, KYSE70 and KYSE450) were cultured at 37°C with 5% CO2 in RPMI-1640 (Gibco, Rockville, USA) supplemented with 100 U/ml penicillin, 100 μg/ml streptomycin and 10% fetal bovine serum (Hyclone laboratories, Logan, USA).

### Small interfering RNA

Small interfering RNA (siRNA) against MALAT1 and negative control with no definite target were employed and synthesized by Shanghai Jikai Gene Chemistry Co. Briefly, transfection reagent (Invitrogen, USA) was incubated with 0.8 ml of serum-free medium for 10 min. Subsequently, siRNA mixture was added to the medium mentioned above. After incubation for 15 min, the mixture was added to TE7 cells, then seeded in 6-well plates for 24 hr to 60% confluence in medium without antibiotics at siRNA concentration of 100 nM and continued to incubate for 6 hr. 1 ml medium containing 10% FBS was added to each well without removing the transfection mixture. New medium was added to the cells after being cultured for 24 hr. The interfering efficiency was determined by qRT-PCR after transfection 48 hr.

### Construction of MALAT1 shRNA vector

To knock down MALAT1 expression, we used pGV248 vector encoding a small hairpin RNA directed against the target MALAT1 in TE7 cells. The target sequence for MALAT1 was 5′-GAGGTGTAAAGGGATTTAT-3′. As a negative control, we used shRNA vector without hairpin oligonucleotides. Lentiviral particles were produced by pGV248-MALAT1 shRNA with viral particle packaging helper vector into 293T cells. The efficiency of knockdown was determined by qRT-PCR. TE7 cells were transfected with the lentivirus with the pGV248-MALAT1 shRNA or pGV248-NC. After 72 hr, transfected TE7 cells were sorted by staining with anti-EGFP Ab using Moflo XDP (Beckman, USA).

### Cell proliferation and cell apoptosis assay

Cells seeded in 96-well plates (5000/well) were transfected with si-MALAT1 or NC, and the cell proliferation assays were conducted every 24 hr using CCK-8 (Djingo, Japan) according to the manufacturer' s protocol. 10 μl CCK-8 dye was added to each well. The number of viable cells was quantified by the absorbance at 450 nm at indicated time points (24 hr, 48 hr and 72 hr) using a microplate reader.

Cells were harvested and stained using an annexin V-FITC apoptosis detection kit according to the manufacturer's instructions. Stained cells were analyzed immediately by Flow Cytometry. Annexin V-FITC at final concentration of 1 μg/ml and 250 ng of Propidium Iodide (PI) was added to a mixture containing 100 μl of cell resuspension and binding buffer each. After cells were vortexed and incubated for 15 min at room temperature (RT) in the dark, 400 μl of binding buffer were added. Cells were analyzed by FACS Calibur and Flowjo software (BD Bioscience, USA). Cells were discriminated into viable cells, dead cells, early apoptotic and late apoptotic cells, relative ratios of the early and late apoptotic cells were compared to the control group.

### Sphere formation assay

TE7 cells transfected with si-MALAT1 or NC was plated on 24-well plates (Corning Costar, USA) at a density of 500/well maintained in RPMI-1640 medium containing 10% FBS and 4 μg/ml heparin (Sigma, USA), 1:50 B27 (Gibco, Rockville, USA), 20 ng/ml EGF, 20 ng/ml basic FGF (Bothfrom, USA), 100 IU/ml penicillin and 100 μg/ml streptomycin for 7 days. The colonies were counted under a low magnification microscope (Leica, Germany) and a group of more than 10 cells was defined as a colony.

### Transwell assay

According to the manufacturer's instruction, transwell assay was performed with 24-well inserted plates (5 μm, Corning Incorporated, USA). 1×10^6^ TE7 cells were added to the upper chamber and 600 μl medium were added in the lower chamber. The plates were incubated at 37°C in a humidified atmosphere of 5% CO2 and banned at the appropriate time. The number of cells migrated to the lower chamber was counted.

### Western blots

Cells protein extraction was prepared with lysis buffer, and protein concentration was determined using Bradford method. The total cellular protein extraction was separated on 10% SDS-PAGE. Proteins were transferred to nitrocellulose membranes by a semi-dry transferor. The membranes were incubated in Tris-buffered saline, and then incubated at RT for 2 hr with antibodies to β-catenin, E-cadherin, Lin28, OCT4, Ezh2 and β-actin, respectively. The corresponding secondary antibodies were used at a 1:1000 dilution. Finally, antibody complex was detected by enhanced chemiluminescence according to manufacturer's protocol.

### In vivo assay

Ten nude mice were purchased from Chinese Academy of Science Shanghai Experimental Animal Center. Mice were maintained with sterilized food and water. Nude mice with 6 weeks old were used for the following experiments. Each mouse was injected subcutaneously with tumor cells (3×10^6^ in 100 μl of medium). Mice were randomized divided into two groups, TE7 cells transfected with MALAT1-shRNA group and negative control group. The body weights of mice and tumor volume were examined every three days. After 15 day, all mice were sacrificed and tumor tissues were weighed.

### Immunohistochemistry

The paraffin-embedded tissues (tumor tissues from nude mice) were examined for the expression of β-catenin and Ezh2. Sections were treated with 3% H_2_O_2_ and 5% BSA and incubated with primary antibodies overnight at 4°C. After incubation with HRP-conjugated secondary antibody for 1 hr at 37°C, sections were washed and counterstained with hematoxylin and visualized under a microscope (Olympus, Japan).

### Study ethics approval

All protocols were approved by the Research Ethics Committee of Zhengzhou University. Written informed consent was obtained from all patients and healthy volunteers.

### Statistical analysis

All data were performed with one-way analysis of variance using Graph Pad software (Prism 5.0). Correlations between the MALAT1 and Ezh2 expression and pathological parameters were evaluated using cross-tabulation. Data from surgical resection to death of the patients were analyzed using the Kaplan-Meier survival curve. The difference between paired groups was measured using the paired *t* test. *P* value less than 0.05 was considered statistically difference (*P<0.05, ** *P*< 0.01, *** *P*< 0.001) and all *P* values were two-sided.

## SUPPLEMENTARY FIGURES


