# Supplementary material for: Long noncoding RNA MALAT1 promotes malignant development of esophageal squamous cell carcinoma by targeting β-catenin via Ezh2
Source: Oncotarget. 2016 Mar 22;7(18):25668–82. doi: 10.18632/oncotarget.8257 (PMC5041935; doi:10.18632/oncotarget.8257)
Supplement: Supplementary file 1 [file oncotarget-07-25668-s001.pdf]

SUPPLEMENTARY FIGURES

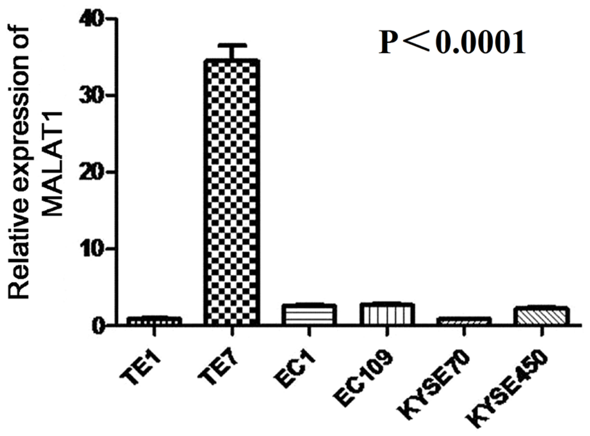

Supplementary Figure S1: The expression analysis of MALAT1 in six ESCC cell lines. qRT-PCR was used to detected the expression of MALAT1 in six esophageal cancer cell lines TE1, TE7, EC1, EC109, KYSE70 and KYSE450. Data are representative of three independent experiments and represent the mean ± SD.

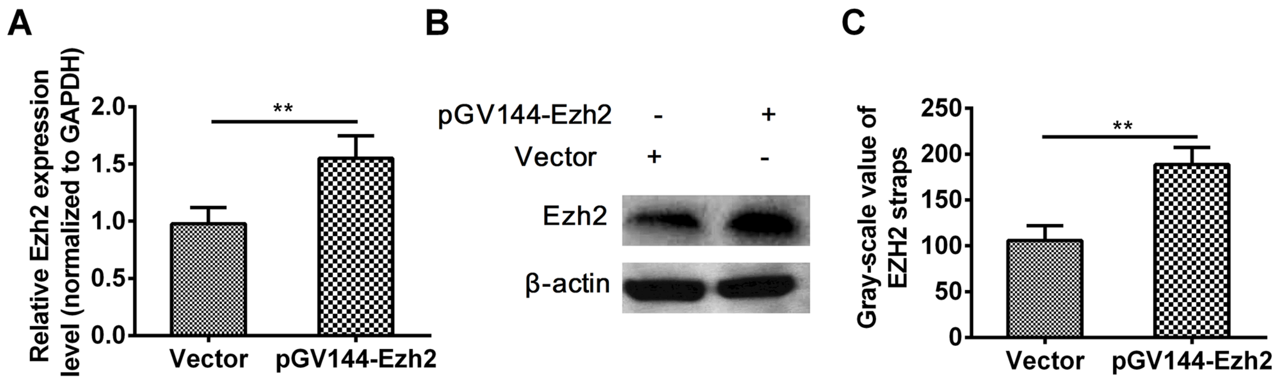

Supplementary Figure S2: Ezh2 expression was evaluated in TE7 cells transfected with pGV144-Ezh2. A. Ezh2 expression was evaluated in TE7 cells transfected with pGV144-Ezh2 or vector measured by qRT-PCR. **B.** Ezh2 expression level was evaluated in TE7 cells transfected with pGV144-Ezh2 or vector measured by Western blots. **C.** Western blots data are representative of three independent experiments and represent the mean ± SD. *, $P<0.05$, **, $P< 0.01$, ***, $P< 0.001$.
